# Localized Facial Bullous Eruption Following Iodinated Contrast in a Patient With Renal Failure

**DOI:** 10.7759/cureus.110513

**Published:** 2026-06-09

**Authors:** Jared R Zhang, Annie Cherner, Mitchell W Cox, Christine L Shokrzadeh

**Affiliations:** 1 General Surgery, The University of Texas Medical Branch at Galveston, Galveston, USA; 2 Vascular Surgery, The University of Texas Medical Branch at Galveston, Galveston, USA

**Keywords:** chronic kidney disease (ckd), contrast allergy, iodinated contrast, iododerma, suspected drug eruption

## Abstract

Iodinated contrast media (ICM) can induce delayed hypersensitivity reactions, but localized recurrent bullous eruptions are exceedingly rare. We present the case of a 51-year-old male with end-stage renal disease (ESRD) who developed a recurrent, highly localized bullous facial dermatosis following repeated intravenous ICM administration for vascular interventions. His initial reaction of diffuse facial flushing evolved with subsequent exposures into severe, anatomically fixed facial blistering that strictly spared the oral mucosa, ultimately resolving into profound post-inflammatory hyperpigmentation. This case presents a unique diagnostic overlap: the strict anatomic recurrence represents the clinical hallmark of a fixed drug eruption, while the severity of the blistering in the setting of ESRD suggests a synergistic toxicokinetic mechanism akin to iododerma. The patient's profound renal impairment likely caused delayed contrast clearance, resulting in a massive systemic iodine load that amplified the hapten-driven localized inflammatory response. Clinicians must recognize evolving hypersensitivity patterns to ICM, as delayed clearance in renal dysfunction can drastically exacerbate localized T-cell-mediated cutaneous reactions, leading to severe dyspigmentation if the offending agent is not promptly identified and avoided.

## Introduction

Iodinated contrast media (ICM) are widely utilized in contemporary diagnostic and interventional medicine to enhance imaging quality. While immediate IgE-mediated anaphylactoid reactions are well-documented, non-immediate delayed hypersensitivity reactions present a more subtle clinical challenge [1]. Delayed hypersensitivity to radiocontrast can manifest from three hours up to several days post-exposure and displays significant morphologic variability [2]. These delayed eruptions are primarily T-cell-mediated, and their pathophysiology often mimics severe cutaneous adverse reactions [3]. Among these delayed reactions, fixed drug eruptions (FDEs) and iododerma are rare and frequently misdiagnosed. FDE is a non-immediate hypersensitivity reaction that typically recurs at identical anatomical sites upon re-exposure to the causative agent, mediated by tissue-resident memory T cells that remain localized in the previously affected epidermis [4]. In contrast, iododerma is a rare cutaneous eruption following iodine administration. While its primary pathogenesis is unclear, it is strongly associated with an immunologic mechanism triggered by delayed iodine clearance, which is almost exclusively driven by renal insufficiency [5]. This report describes the case of a patient with end-stage renal disease (ESRD) who developed a recurrent, strictly localized bullous facial dermatosis that progressed to severe post-inflammatory hyperpigmentation (PIH) after multiple intravenous ICM exposures.

## Case presentation

The patient is a 51-year-old male with a complex medical history, including ESRD managed with hemodialysis three times a week, heart failure with reduced ejection fraction (30-35%), and multivessel coronary artery disease requiring prior interventions. His baseline serum creatinine historically ranged from 3.5 to 5.0 mg/dL. The initial hypersensitivity event occurred in March 2025. After a high-risk percutaneous coronary intervention involving substantial contrast administration, the patient experienced severe facial flushing, edema, pruritus, and mild throat tightness while in the recovery unit approximately seven hours post-procedure. The patient was acutely managed for a suspected severe allergic reaction with intravenous diphenhydramine and methylprednisolone. Due to the severity of the facial edema, he was transferred to the coronary care unit for close monitoring and subsequently managed with an intravenous dexamethasone taper and oral cetirizine, resulting in clinical resolution within 24 hours. Allergy and immunology consultation suspected a delayed hypersensitivity reaction to the contrast media and recommended strict avoidance.

Despite this recommendation, repeated angiographic and endovascular procedures were necessary in the following months to address severe vascular comorbidities, including a right upper extremity arteriovenous fistula revision for steal syndrome. After these subsequent exposures to contrast media, the patient reported reproducible episodes of intense facial pruritus accompanied by the acute formation of tense, fluid-filled bullae. These recurrent blistering episodes remained strictly confined to the face, with complete sparing of the oral mucosa. The cutaneous lesions were not treated with systemic immunosuppression during these outpatient exposures and resolved with epidermal desquamation over several weeks.

**Figure 1:**
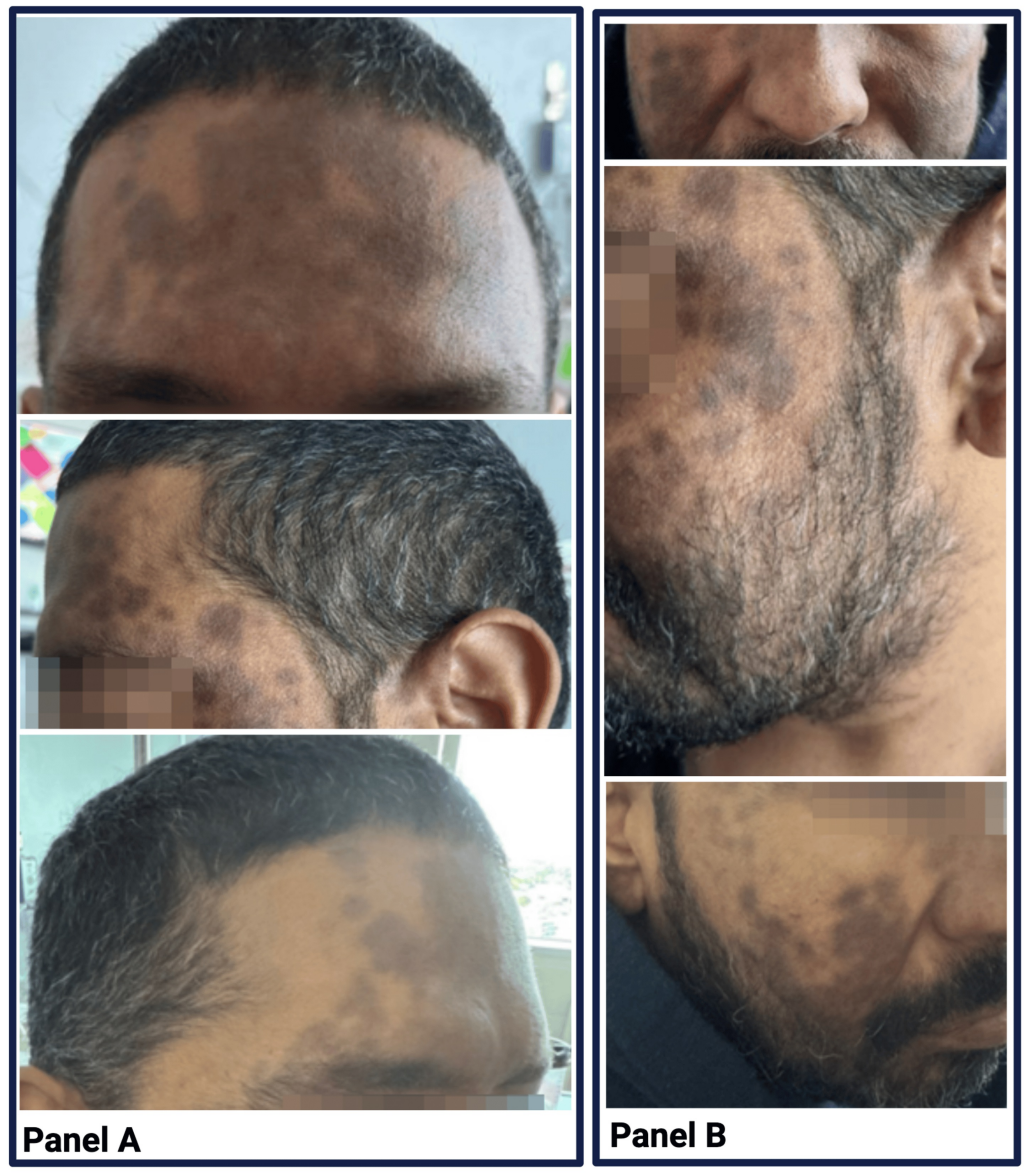
Extensive facial post-inflammatory hyperpigmentation following the resolution of an acute localized bullous dermatosis Panel A: close-up view of the forehead demonstrating irregular, coalescing hyperpigmented macules. Panel B: lateral view of the cheek highlighting the sharp demarcation of the hyperpigmented patches and complete sparing of the mucosal surfaces.

In October 2025, the patient was admitted for the evaluation and management of severe lower extremity complications, specifically a left foot wound and a persistent right foot ulcer. During this admission, his renal impairment was profound, with serum creatinine peaking at 8.88 mg/dL. Dermatology was consulted to evaluate prominent facial scars and hyperpigmentation, prompted by a "rash after contrast." On dermatologic examination, the acute blistering phase had resolved, revealing scattered hyperpigmented macules and patches that had coalesced extensively across the face (Figure 1).

Clinical assessment determined that the presentation was consistent with PIH following a recent exfoliative or bullous rash. The clear temporal association between interventional procedures and the onset of localized pruritus and bullae indicated that the primary dermatosis was secondary to a contrast allergy. The dermatologic treatment course focused on conservative management, emphasizing strict, absolute avoidance of all contrast media to prevent recurrence. The patient was counseled that the PIH would fade gradually over several months and was instructed on a daily regimen of diligent photoprotection utilizing SPF 30+ sunscreen.

## Discussion

This case demonstrates a profound, recurrent, and morphologically specific delayed hypersensitivity reaction to ICM. The progression from diffuse facial flushing to a recurrent, highly localized blistering eruption with severe PIH presents a unique diagnostic overlap. The clinical course reflects an intersection between the localized memory of an FDE and the toxicokinetic accumulation characteristic of iododerma, both amplified by underlying ESRD.

The clinical features of the rash are highly suggestive of a bullous FDE secondary to iodinated contrast. As described by Mota et al., FDE is a delayed hypersensitivity reaction that recurs at the same anatomical sites upon re-exposure to the causative agent [6]. The recurrent bullae forming strictly and repeatedly on the face after successive angiographic procedures in this case mirrors the pathophysiologic hallmark of strict anatomical recurrence via tissue-resident memory T cells. Furthermore, the complete sparing of the oral mucosa helps differentiate this highly localized process from broader, potentially life-threatening mucocutaneous syndromes. While patch testing within previously affected areas remains the gold standard diagnostic tool for confirming T-cell-mediated hypersensitivity to specific ICM agents, the acute inflammatory phase had already transitioned to PIH by the time of dermatologic evaluation in this patient, precluding this diagnostic step [6,7].

While a T-cell-mediated FDE accounts for the anatomical restriction of the rash, it does not fully explain the severity of the bullous presentation without considering the significant pharmacokinetic consequences of renal failure. Iododerma is a rare cutaneous reaction resulting from systemic iodine accumulation. Rothman et al. documented a case of iododerma following serial CT scans, noting that although it can occur in patients with relatively normal renal function, renal insufficiency is a primary risk factor because it severely delays iodine clearance [8]. The exact underlying mechanism relies on an immunologic process; accumulated iodine promotes adverse reactions in susceptible individuals through delayed-type hypersensitivity via haptenization [5]. A review of renal function during the index October 2025 hospitalization demonstrates this profound pharmacokinetic vulnerability. This patient had ESRD with a baseline creatinine of approximately 4.5 mg/dL, peaking at 8.88 mg/dL. Importantly, he was maintained on his standard thrice-weekly hemodialysis schedule without augmented immediate post-procedural clearance. Because ICM is almost exclusively renally excreted, severe impairment combined with unadjusted dialysis intervals resulted in prolonged systemic retention of contrast media after high-volume vascular procedures [9]. This substantial iodine load significantly amplified the hapten-driven localized inflammatory response. Classic iododerma typically presents as papulopustular or vegetative lesions in a seborrheic distribution, which closely aligns with the strictly facial presentation observed in this case [8].

The management of complex delayed contrast reactions requires an adaptive, stage-appropriate therapeutic approach. While aggressive systemic therapies are often employed during acute phases, severe systemic contrast-induced rashes may exhibit unpredictable responses to systemic corticosteroids [10]. El Hussein et al. described a case in which hypersensitivity to intravenous iodine resulted in multiple evolving rash presentations in a patient with acute kidney injury who proved completely refractory to standard intravenous corticosteroids [10]. In their report, marked improvement was achieved rapidly through the use of high-potency topical steroids alone [10]. This paradigm shift demonstrates that even complex contrast reactions in the context of renal impairment may respond well to localized, topically targeted immunomodulation. In the presented case, the acute blistering phase had entirely resolved into PIH by the time of dermatologic evaluation. Therefore, management appropriately omitted aggressive systemic pharmacotherapy. The clinical approach correctly focused on complete withdrawal of iodine exposure, as failure to remove the iodine source in severe iododerma can be fatal [11]. Strict avoidance across all ICM classes is recommended for this patient, as there is documented and unpredictable cross-reactivity among various iodinated contrast agents [12].

## Conclusions

A primary limitation in the diagnostic workup was the absence of a skin biopsy during the acute bullous phase. Histopathological examination could have definitively distinguished the pseudoepitheliomatous hyperplasia and diffuse neutrophilic dermal infiltrate characteristic of iododerma from the interface dermatitis and melanophage presence typical of FDE. Additionally, exact cumulative contrast volumes (in mL) and acute-phase inflammatory markers (e.g., eosinophil counts) were unrecorded during the outpatient procedures, and the lack of patch testing prevented definitive identification of the specific offending agent.

Intravascular ICM can provoke severe, delayed hypersensitivity reactions that worsen with repeated exposures. This case likely represents a hybrid phenomenon: a localized FDE with clinical severity amplified into an iododerma-like bullous eruption by toxic accumulation of contrast media due to ESRD. Clinicians managing complex vascular patients should maintain a high index of suspicion for recurrent bullous eruptions when localized cutaneous symptoms occur after imaging. Strict avoidance of the contrast agent and timely, stage-appropriate dermatologic intervention are essential to prevent recurrent blistering and permanent dyspigmentation.
